# Evaluating Self-Management Behaviors of Diabetic Patients in a Telehealthcare Program: Longitudinal Study Over 18 Months

**DOI:** 10.2196/jmir.2699

**Published:** 2013-12-09

**Authors:** Lichin Chen, Lee-Ming Chuang, Chia-Hsiun Chang, Chiou-Shiang Wang, I-Ching Wang, Yufang Chung, Hui-Yu Peng, Hui-Chuen Chen, Yu-Ling Hsu, Yu-Sheng Lin, Huang-Jen Chen, Tieng-Chun Chang, Yi-Der Jiang, Hung-Chang Lee, Ching-Ting Tan, Hsin-Lu Chang, Feipei Lai

**Affiliations:** ^1^Graduate Institute of Biomedical Electronics and BioinformaticsNational Taiwan UniversityTaipeiTaiwan; ^2^Department of Internal MedicineNational Taiwan University HospitalNational Taiwan UniversityTaipeiTaiwan; ^3^Department of NursingNational Taiwan University HospitalNational Taiwan UniversityTaipeiTaiwan; ^4^Department of Electrical EngineeringTunghai UniversityTaichungTaiwan; ^5^Department of DieteticsNational Taiwan University HospitalNational Taiwan UniversityTaipeiTaiwan; ^6^Department of Computer Science and Information EngineeringNational Taiwan UniversityTaipeiTaiwan; ^7^Department of Information ManagementTamkang UniversityTaipeiTaiwan; ^8^Department of OtolaryngologyCollege of MedicineNational Taiwan UniversityTaipeiTaiwan; ^9^Departmanet of Management Information SystemsNational Chengchi UniversityTaipeiTaiwan; ^10^Department of Electrical EngineeringNational Taiwan UniversityTaipeiTaiwan

**Keywords:** Internet, diabetes mellitus, telemedicine, self-care, online systems, personal health record, patient access to records

## Abstract

**Background:**

Self-management is an important skill for patients with diabetes, and it involves frequent monitoring of glucose levels and behavior modification. Techniques to enhance the behavior changes of diabetic patients have been developed, such as diabetes self-management education and telehealthcare. Although the patients are engaged in self-management activities, barriers to behavior changes remain and additional work is necessary to address the impact of electronic media and telehealthcare on patient self-care behaviors.

**Objective:**

The aims of this study were to (1) explore the behaviors of diabetic patients interacting with online applications, (2) determine the impact of a telehealthcare program among 7 self-care behaviors of the patients, and (3) determine the changes in glycosylated hemoglobin (HbA_1c_) levels.

**Methods:**

A telehealthcare program was conducted to assist the patients with 7 self-care activities. The telehealthcare program lasted for 18 months and included the use of a third-generation mobile telecommunications glucometer, an online diabetes self-management system, and a teleconsultant service. We analyzed the data of 59 patients who participated in the telehealthcare program and 103 who did not. The behavioral assessments and the HbA_1c_ data were collected and statistically analyzed to determine whether the telehealthcare services had an impact on the patients. We divided the 18-month period into 3 6-month intervals and analyzed the parameters of patients assisted by the telehealthcare service at different time points. We also compared the results of those who were assisted by the telehealthcare service with those who were not.

**Results:**

There was a significant difference in monitoring blood glucose between the beginning and the end of the patient participation (*P*=.046) and between the overall period and the end of patient participation (*P*<.001). Five behaviors were significantly different between the intervention and control patients: being active (*P*<.001), healthy eating (*P*<.001), taking medication (*P*<.001), healthy coping (*P*=.02), and problem solving (*P*<.001). Monitoring of blood glucose was significantly different (*P*=.02) during the 6-12 month stage of patient participation between the intervention and control patients. A significant difference between the beginning and the 6-12 month stage of patient participation was observed for the mean value of HbA_1c_ level (*P*=.02), and the differences between the overall HbA_1c_ variability and the variability of each 6-month interval was also significant.

**Conclusions:**

Telehealthcare had a positive effect on diabetic patients. This study had enhanced blood glucose monitoring, and the patients in the program showed improvements in glycemic control. The self-care behaviors affect patient outcomes, and the changes of behavior require time to show the effects.

## Introduction

### Background

Self-management is an important skill for patients with diabetes mellitus [1-3], involving frequent monitoring of glucose levels and behavior modification. The primary goal of self-management is to monitor glucose metabolism and induce behavioral changes to achieve better glycemic control [3,4]. However, some patients do not respond to behavior modification and some do not have sufficient knowledge to perform self-management [5-9]. Several techniques to enhance self-care behaviors for diabetic patients have been developed, such as diabetes self-management education (DSME) and telehealthcare. DSME provides patients with self-management skills, supports behavioral changes, and achieves optimal patient outcomes in diabetes care [10-13]. Telehealthcare programs promote preventive care, self-management, and clinical consultations from a distance [6,7,9,14-16]. However, self-management is challenging because it requires behavioral changes and can be easily neglected owing to a busy lifestyle and lack of support. Barriers remain for patients who carry out self-care tasks. Previous studies have also highlighted the need for additional research to address the impact of telehealthcare and electronic media on patient self-care behaviors [17,18].

In addition to routine DSME, patients in the present study completed a telehealthcare program using a third-generation mobile telecommunication (3G) glucometer, an online diabetes self-management system, and a teleconsultant service. The aim of the present study was to assess how diabetic patients used the online diabetes self-management system, determine the impact of a telehealthcare program among the 7 self-care behaviors, and measure the changes in glycosylated hemoglobin (HbA_1c_) level. The 7 self-care behaviors are based on the definition of the American Association of Diabetes Educators 7 Self-Care Behaviors (AADE7).

### Diabetes Care in Taiwan

As effective management for chronic diseases has been established, the National Health Insurance (NHI) system of Taiwan has acknowledged the value of disease screening and disease management. The NHI has broadened payment for several disease-management programs and provided financial incentives for regular diabetes follow-up visits [19-23]. In 2001, a diabetes pay-for-performance program, known as the diabetes shared care network, was implemented [24-26]. It emphasizes the value of a multidisciplinary care team to provide DSME and regular screening to enhance self-management skills and the early detection of complications [13].

Patients are required to return for a regular visit every 3 months for laboratory monitoring and DSME courses. Certified diabetes educators (CDEs) educate patients and evaluate their self-care behaviors and skills, which is documented in their behavioral assessments. Using past medical records and laboratory results, the CDEs attempt to determine patient self-management problems. After the patients demonstrate capability in a behavior, the CDEs proceed to the next behavior. The CDEs continue this process until all potential changes are made to achieve better metabolic control. When the patients actively provide information and ask questions about self-management, the CDEs are likely to provide additional education to address the patients’ needs. Typically, the CDEs provide education on and evaluate 1 to 2 behaviors per visit. The number and the distribution of the behavioral assessments are an indicator of underlying patient problems. It is, therefore, meaningful to explore the patterns of and changes in patient problems to provide more adequate health care support.

### Diabetes Care at National Taiwan University Hospital

In 2001, the shared care network was implemented at the National Taiwan University Hospital (NTUH), a 2300-bed educational medical center in Taiwan. Currently, the DSME and assessments are documented in the disease management information system (DMIS), developed in 2011 [27]. The DMIS is based on the AADE7 framework and includes the following items: healthy eating, being active, monitoring, taking medication, problem solving, healthy coping, and reducing risks. The detailed contents of the AADE7 are shown in Multimedia Appendix 1. In addition to the shared care network, a telehealthcare program was initiated in 2011 to explore the effectiveness of information technology interventions for individuals with diabetes.

Telehealthcare allows for the promotion of health care services outside the medical institute. Through the combination of information technology and commercial biosensor devices, telehealthcare facilitates longitudinal health status monitoring at a distance [5,14,28,29]. The effectiveness of telehealthcare has been indicated by the industry and policymakers [29-34], and online informatics applications have been widely adopted in diabetes care [1,6,35-37]. It is a promising technology for self-management and can increase patient knowledge, patient engagement, condition monitoring, long-term follow-ups [1,7,8,16,38], and improved patient outcomes [15,16,38,39].

## Methods

### Overview

In addition to the routine care of individuals with diabetes mellitus, this research provided a telehealthcare program and aimed to illustrate the way patients used and interacted with the online diabetes self-management system, the impact of a telehealthcare program among 7 self-care skills, and changes in patients’ HbA_1c_ levels. A diabetes telehealthcare program was conducted for 18 months, wherein patients received assistance from an online diabetes self-management system to record and manage their daily activities, a 3G glucometer to monitor their glucose, and a teleconsultant service to enhance patients’ self-management activities. Behavioral assessments and HbA_1c_ levels were documented in the DMIS. We compared these parameters for those who were assisted by the telehealthcare service (intervention group) at different time points. We also compared the intervention group results to those of patients who did not participate in the telehealthcare program (control group).

### The Online Diabetes Self-Management System and Teleconsultant Service

The design of the online diabetes self-management system was based on personal health record (PHR) criteria. The PHR is a health record in which patients’ health data and personal information are recorded and maintained by the patients themselves [40]. It is owned, controlled, and managed by the patients [41-43], can be easily accessed, is not limited to operating systems or devices [41-44], ensures interoperability of data between diverse systems [41,42,44-47], and facilitates easy ways of uploading information [42,43,48]. The PHR emphasizes patient privacy, providing adequate data encryption and a secured environment [38,41-44]. In addition, it improves communication and information sharing between patients and care providers [1,6,41-44,48].

**Figure 1:**
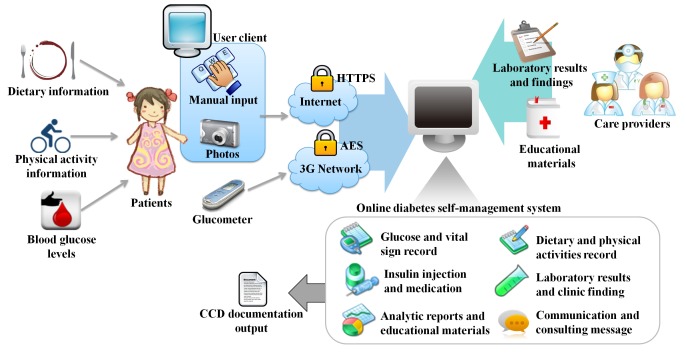
Infrastructure design of the online diabetes self-management system. Patients record their daily activities through manual input, uploading photographs, and a 3G glucometer. The data are transmitted to the system through hypertext transfer protocol secure (HTTPS) and advanced encryption standard (AES) encryption. Caregivers can also enter the system and provide information and support. The system includes information to support self-management. The data can be generated in continuity of care document (CCD) format to ensure the interoperability between systems.

The online diabetes self-management system was developed using C# programming language and a Microsoft SQL server. It was integrated with an off-the-shelf 3G glucometer [14]. The infrastructure design of the online diabetes self-management system is shown in Figure 1. According to the scheme mentioned above, each patient is assigned a unique identification and password, which allows them to log in, edit, and manage their information. The online diabetes self-management system is a Web-based system accessible on the Internet and not limited to operating systems or devices. It adopted the continuity of care document (CCD) standard to enhance the interoperability of data. The 3G glucometer facilitated easy and automatic data uploading after each measurement anywhere and combined blood pressure, blood glucose, and heart rate measurements in 1 instrument.

Encryptions were used to ensure patient privacy and for data transmission. The Web connection between a user client and the server was encrypted using the hypertext transfer protocol secure (HTTPS) protocol, and data transmission between the glucometers and the server was enciphered with advanced encryption standard (AES) encryption. Asynchronous JavaScript and XML (Ajax) and JQuery JavaScript were used to validate the format of the data input and to cooperate with different browsers.

 Asynchronous text messages were provided in the online diabetes self-management system; patients and caregivers could communicate through the online diabetes self-management system internal message service or short message service (SMS) text messaging. Application programming interfaces (API) from the Internet were used to send and retrieve information such as weather and pollution standard indexes. The online diabetes self-management system exchanged data with the hospital information system (HIS) using a service-oriented architecture (SOA) mechanism. The Health Level 7 (HL7) embedded Extensible Markup Language (XML) formatted data were used in the framework for data exchange. Patients were able to see their blood test results from the online diabetes self-management system.

The online diabetes self-management system included the monitoring items and the diabetes-related information, such as blood glucose, blood pressure, heart rate, body weight, insulin injection, daily diet, and daily physical activities. Information that was measured with equipment that did not have transmission networks required manual input. Dietary intake could be recorded through the use of either text or images. Additional information to enable self-management and goal setting for glucose control were generated (eg, the mean, median, standard deviation, and maximum and minimum daily blood glucose values). The variations in blood glucose and other parameters are presented together graphically to enable the user to observe the effect of each behavior. The frequency of self-monitoring of blood glucose (SMBG) was recorded and compared with the set goals to determine whether adjustments were needed. Body mass index (BMI) was calculated, and the suggested calorie intake and ingredient volume for each meal were displayed. An additional care-provider interface was designed so that caregivers could get a quick overview of patient status. Case managers were able to log in and view the data uploaded by the patients, identify abnormal events, and make phone calls. The online diabetes self-management system sent an SMS text message to care providers when the data exceeded the alerting range.

This study provided a teleconsultant service to support patients with diabetes self-management. The case managers for this study, including a nurse and a dietitian, were the care providers who interacted with the patients from a distance. They were responsible for monitoring patient status, answering questions about self-care activities, regularly keeping in touch with the patients through telephone calls or text messages, and encouraging them to perform self-management. The care plans and goal setting were formulated through a discussion with each patient during his or her enrollment. The case managers monitored the data uploaded by the patients. They gave advice and reminded the patients to perform self-care activities. In this study, the case managers were not involved in medication adjustments. They did, however, collate patient data and bring the information to the clinic when the patient returned for an appointment. They communicated with physicians to suggest adjustments when needed.

The assistance of the online diabetes self-management system and the teleconsultant service covered the 7 behaviors of self-management activities (shown in Multimedia Appendix 1). During the study period, the CDEs did not know whether the patient attended the telehealthcare program.

### Patient Enrollment and Data Analysis

Candidates for the study included patients diagnosed with either Type I diabetes mellitus (T1DM) or Type II diabetes mellitus (T2DM) and those with an HbA_1c_ level greater than 7.5 or identified as not well controlled. Patients with severe diabetes complications, such as diabetic foot, diabetic proliferative retinopathy, liver dysfunction, end-stage renal disease, or other medical problems that could affect the study results or trial participation were excluded. The patients were approached during regular follow-up visits in their physician’s office, and informed consent was obtained from each participant. After enrollment, patients were taught to use the online diabetes self-management system and the glucometer, and they were informed how to contact the case manager for assistance. The glucometer and test strips were provided for glucose monitoring without a charge. Patients were allowed to use their own glucometer if they preferred. Those who chose not to use the provided glucometer were allowed to input their data manually. This study was reviewed and approved by the NTUH Institutional Review Board (IRB) (No. 201108018RC).

By the end of February 2013, 184 patients were enrolled in the telehealthcare program. Of the patients who participated in the telehealthcare program, 59 (32.1%) were also in the shared care network. The results of participants in the telehealthcare and shared care network were also compared to those of the participants who did not use the telehealthcare service but did complete the behavioral assessments and laboratory monitoring; these individuals served as the control participants. We recruited 103 control participants and matched their demographic characteristics with those of the telehealthcare participants to minimize the effect of potential confounding variables, including gender, age, diabetes type, duration, years of participation in the shared care network, and insulin treatment. We focused on analyzing the data of patients in the telehealthcare and the control groups, who were evaluated by behavioral assessments and laboratory monitoring every 3 months.

**Figure 2:**
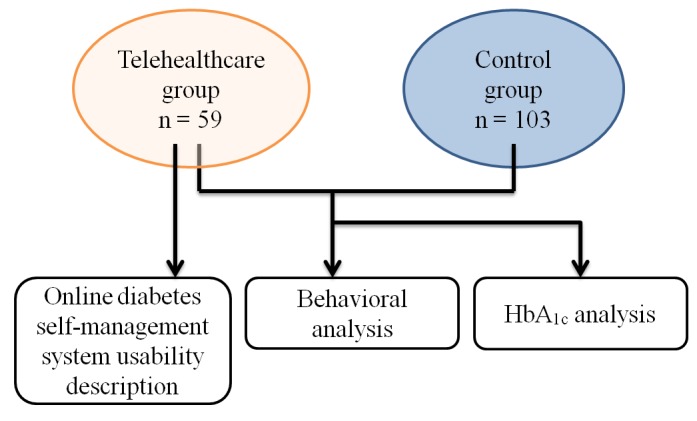
A flowchart of the data analysis.

The evaluation of this study consisted of 3 sections (shown in Figure 2). We first analyzed the logged records of the system and illustrated the way patients used the online diabetes self-management system. For the second and third sections, the behavioral assessments and laboratory results from the DMIS for 18 months were evaluated by within-group comparisons at different time points for those participants who entered the telehealthcare program. We also compared the telehealthcare and control groups at different time points.

**Figure 3:**
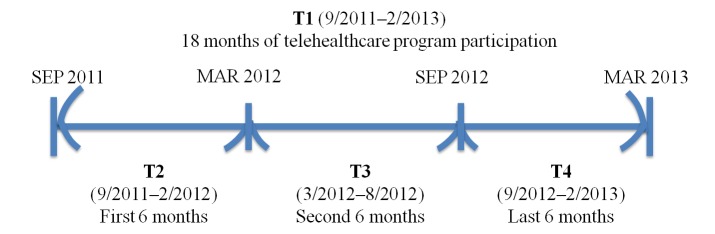
Analysis timeline.

The data were grouped into several time ranges (see Figure 3). The time ranges included the entire 18 months of patient participation (September 2011 to February 2013, T1), the first 6 months (September 2011 to February 2012, T2), the second 6 months (March 2012 to August 2012, T3), and the last 6 months of patient participation (September 2012 to February 2013, T4). The data were grouped in 6-month intervals to obtain 1 or 2 records from regular patient visits.

The second section was the behavioral analysis. The number and distribution of the behavioral assessments indicated the problems of patients while performing self-management. In the behavioral analysis, the number of behavioral assessments was calculated for the 7 behaviors for each time range. The number of behavioral assessments of the telehealthcare participants during T1 was compared with each range (T2, T3, and T4) to demonstrate the overall variation of each time range, and the baseline (T2) was compared with T3 and T4 to see the variation across time. The differences between the telehealthcare and the control participants at each range (T1, T2, T3, and T4) were also compared.

The third section was the HbA_1c_ analysis, which consisted of calculating the HbA_1c_ mean and analyzing HbA_1c_ variability. HbA_1c_ variability represents intraindividual differences for each patient, which refers to the changes in glycemia over longer periods of time reflected in changes in HbA_1c_ from one visit to the next [49,50]. It is defined as the standard deviation (SD) of the serial HbA_1c_ measurements. All data for blood tests within each time range were collected, and the HbA_1c_ mean and HbA_1c_ variability were calculated for each patient during each time range. The value for the telehealthcare participants at each time range (T2, T3, and T4) was compared to T1 to observe the overall variation of each time range, and the changes at T3 and T4 were observed by comparing to their baselines (T2). The differences between the telehealthcare and control participants at each time range (T1, T2, T3, and T4) were compared.

Paired *t* tests were used to analyze the differences in variables of interest between the time points of the telehealthcare participants’ measures. Independent *t* tests were used to compare the telehealthcare and control results and between T1 and each time range for telehealthcare participants. We used SPSS version 17.0 (SPSS Inc, Chicago, IL, USA) for the statistical analyses.

## Results

**Figure 4:**
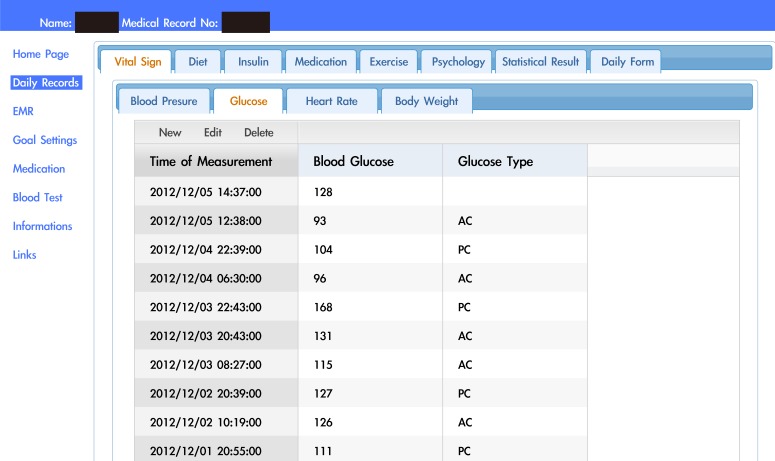
Screenshot of the online diabetes self-management system user interface (note that the original user interface is in Chinese).

Table 1 and Multimedia Appendices 2-4 show the demographic information of the telehealthcare and control participants, including gender, age, years of participation in the shared care network, disease duration, and the use of insulin injection. The telehealthcare group consisted of 18 T1DM and 41 T2DM patients. The control group consisted of 32 T1DM and 71 T2DM patients. The average age of the telehealthcare group was 51.34 years (SD 12.79). Figure 4 shows the user interface of the online diabetes self-management system. Overall, 90% (53/59) of the patients logged in and used the online diabetes self-management system. On average, patients logged in 1.3 (SD 2.2) times every week and performed 1.1 (SD 1.3) SMBG daily. Analysis of the self-management records showed that 98% (58/59) of the patients documented blood glucose, 73% (43/59) documented blood pressure, 69% (41/59) documented heart rate, 44% (26/59) documented dietary record, 44% (26/59) documented insulin injections, and 31% (31/59) documented physical activities. In all, 61% (36/59) of the patients used telephone services to contact the case managers; 56% (33/59) used text messages.

Table 2 shows the behavioral assessments of telehealthcare group during different time ranges. SMBG was significantly different between T1 and T4 (*P*<.001) and T2 and T4 (*P*=.046). Most of the number of assessments increased at T3 and decreased at T4, and SMBG was the only behavior that continued to increase until T4. There was a statistically significant difference between the telehealthcare and control participants in 5 behaviors at T1 (Table 3), including being active (*P*<.001), healthy eating (*P*<.001), taking medication (*P*<.001), healthy coping (*P*=.02), and problem solving (*P*<.001). SMBG was significantly different between the telehealthcare and control groups at T3 (*P*=.02). Notably, the telehealthcare participants had more assessments in healthy coping and reducing risk at T4 than did the control participants.

The HbA_1c_ mean and variability differences for the telehealthcare group are shown in Table 4. The mean HbA_1c_ level decreased significantly (*P*=.02) at T3 compared to the baseline (T2) and slightly increased at T4, and the HbA_1c_ mean decreased from 7.8 to 7.68. There was a significant difference in HbA_1c_ variability between T1 and T2 (*P*<.001), T1 and T3 (*P*<.001), and T1 and T4 (*P*<.001), and the HbA_1c_ variability decreased from 0.30 to 0.23 during patient participation. The HbA_1c_ mean and variability differences between the telehealthcare and control groups are shown in Table 5, and were not statistically significant.

**Table 1:** Patient demographics, including gender, age, and diabetes type (N=162).

Demographic information		Telehealthcare n=59	Control n=103
**Gender, n (%)**			
	Male	29 (49.2)	52 (50.5)
	Female	30 (50.8)	51 (49.5)
Age, mean (SD)		51.3 (12.8)	52.55 (12.19)
**T1DM** ^a^			
	<45 years, n (%)	10 (55.6)	23 (71.9)
	>45 years, n (%)	8 (44.4)	9 (28.1)
	Age, mean (SD)	41.78 (9.78)	41.25 (8.16)
**T2DM** ^b^			
	<65 years, n (%)	32 (78.0)	53 (74.6)
	>65 years, n (%)	9 (22.0)	18 (25.4)
	Age, mean (SD)	55.54 (11.73)	57.65 (10.11)

^a^T1DM: Type 1 diabetes mellitus.

^b^T2DM: Type 2 diabetes mellitus.

**Table 2:** The American Association of Diabetes Educators 7 Self-Care Behaviors (AADE7) education of patients with the telehealthcare service (n=59).

Time^a^ and AADE7 behavior		Behavioral assessments, mean (SD)	Comparison with T1, *P* value	Comparison with T2, *P* value
**T1 (n=59)**				
	AADE7 education			
	Being active	0.36 (0.36)		
	Healthy eating	0.60 (0.35)		
	Taking medication	0.51 (0.36)		
	Healthy coping	0.09 (0.22)		
	Problem solving	0.31 (0.36)		
	Reducing risks	0.02 (0.10)		
	Monitoring	0.56 (0.15)		
**T2 (n=59)**				
	Being active	0.34 (0.54)	.66	
	Healthy eating	0.58 (0.59)	.66	
	Taking medication	0.53 (0.60)	.95	
	Healthy coping	0.08 (0.28)	.90	
	Problem solving	0.42 (0.53)	.18	
	Reducing risks	0.00 (0.00)	.10	
	Monitoring	0.63 (0.69)	.68	
**T3 (n=59)**				
	Being active	0.42 (0.56)	.44	.42
	Healthy eating	0.66 (0.63)	.55	.49
	Taking medication	0.54 (0.60)	.71	.89
	Healthy coping	0.10 (0.30)	.82	.66
	Problem solving	0.27 (0.49)	.62	.07
	Reducing risks	0.03 (0.26)	.76	.32
	Monitoring	0.68 (0.71)	.23	.73
**T4 (n=59)**				
	Being active	0.31 (0.60)	.58	.86
	Healthy eating	0.58 (0.60)	.75	.27
	Taking medication	0.46 (0.63)	.59	.90
	Healthy coping	0.08 (0.28)	.90	.90
	Problem solving	0.24 (0.54)	.38	.18
	Reducing risks	0.03 (0.18)	.68	.16
	Monitoring	0.75 (0.63)	<.001	.046

^a^T1: Entire duration of the telehealthcare program, from September 2011 to February 2013 (18 months); T2: initial stage of the telehealthcare program, from September 2011 to February 2012 (6 months); T3: middle stage of the telehealthcare program, from March 2012 to August 2012 (6 months); T4: last stage of the telehealthcare program, from September 2012 to February 2013 (6 months); T4: Last stage of the telehealthcare program, from September 2012 to February 2013 (6 months).

**Table 3:** The American Association of Diabetes Educators 7 Self-Care Behaviors (AADE7) education of patients with and without the telehealthcare service.

Time^a^ and AADE7 behavior		Behavioral assessments per patient, mean (SD)		*P* value
		Telehealthcare (n=59)	Control (n=103)	
**T1**				
	Being active	0.36 (0.36)	0.44 (0.41)	<.001
	Healthy eating	0.60 (0.35)	0.72 (0.43)	<.001
	Taking medication	0.51 (0.36)	0.50 (0.40)	<.001
	Healthy coping	0.09 (0.22)	0.08 (0.17)	.02
	Problem solving	0.31 (0.36)	0.34 (0.37)	<.001
	Reducing risks	0.02 (0.10)	0.02 (0.11)	.29
	Monitoring	0.56 (0.15)	0.60 (0.41)	.94
**T2**				
	Being active	0.34 (0.54)	0.45 (0.61)	.27
	Healthy eating	0.58 (0.59)	0.71 (0.67)	.20
	Taking medication	0.53 (0.60)	0.49 (0.63)	.69
	Healthy coping	0.08 (0.28)	0.08 (0.31)	.91
	Problem solving	0.42 (0.53)	0.45 (0.70)	.84
	Reducing risks	0.00 (0.00)	0.01 (0.10)	.45
	Monitoring	0.63 (0.69)	0.80 (0.74)	.14
**T3**				
	Being active	0.42 (0.56)	0.42 (0.57)	.93
	Healthy eating	0.66 (0.63)	0.71 (0.73)	.65
	Taking medication	0.54 (0.60)	0.52 (0.67)	.87
	Healthy coping	0.10 (0.30)	0.10 (0.30)	.96
	Problem solving	0.27 (0.49)	0.31 (0.50)	.66
	Reducing risks	0.03 (0.26)	0.02 (0.14)	.66
	Monitoring	0.68 (0.71)	0.42 (0.53)	.02
**T4**				
	Being active	0.31 (0.60)	0.48 (0.64)	.11
	Healthy eating	0.58 (0.59)	0.73 (0.70)	.18
	Taking medication	0.46 (0.63)	0.50 (0.61)	.64
	Healthy coping	0.08 (0.28)	0.05 (0.22)	.38
	Problem solving	0.24 (0.54)	0.25 (0.44)	.80
	Reducing risks	0.03 (0.18)	0.02 (0.20)	.66
	Monitoring	0.75 (0.63)	1.69 (0.47)	.13

^a^T1: The entire duration of the telehealthcare program, from September 2011 to February 2013 (18 months); T2: Initial stage of the telehealthcare program, from September 2011 to February 2012 (6 months); T3: Middle stage of the telehealthcare program, from March 2012 to August 2012 (6 months); T4: Last stage of the telehealthcare program, from September 2012 to February 2013 (6 months).

**Table 4:** The HbA_1c_ mean and variability of the patients with the telehealthcare service (n=59).

Time^a^ and HbA_1c_		Mean (SD)	Compared with T1, *P* value	Compared with T2, *P* value
**T1**				
	Mean	7.72 (0.51)		
	Variability	0.51 (0.29)		
**T2**				
	Mean	7.80 (0.38)	.48	
	Variability	0.30 (0.31)	<.001	
**T3**				
	Mean	7.64 (0.40)	.62	.02
	Variability	0.31 (0.31)	<.001	.81
**T4**				
	Mean	7.68 (0.31)	.80	.17
	Variability	0.23 (0.18)	<.001	.11

^a^T1: The entire duration of the telehealthcare program, from September 2011 to February 2013 (18 months); T2: Initial stage of the telehealthcare program, from September 2011 to February 2012 (6 months); T3: Middle stage of the telehealthcare program, from March 2012 to August 2012 (6 months);

**Table 5:** The HbA_1c_ mean and variability of the patients with and without the telehealthcare service.

Time^a^ and HbA_1c_		HbA_1c_, mean (SD)		*P* value
		Telehealthcare (n=59)	Control (n=103)	
**T1**				
	Mean	7.72 (0.51)	7.65 (0.52)	.70
	Variability	0.51 (0.29)	0.52 (0.32)	.78
**T2**				
	Mean	7.80 (0.38)	7.67 (1.45)	.39
	Variability	0.30 (0.31)	0.23 (0.27)	.14
**T3**				
	Mean	7.64 (0.40)	7.67 (1.57)	.89
	Variability	0.31 (0.31)	0.27 (0.26)	.45
**T4**				
	Mean	7.68 (0.31)	7.66 (0.34)	.94
	Variability	0.23 (0.18)	0.25 (0.23)	.46

^a^T1: The entire duration of the telehealthcare program, from September 2011 to February 2013 (18 months); T2: Initial stage of the telehealthcare program, from September 2011 to February 2012 (6 months); T3: Middle stage of the telehealthcare program, from March 2012 to August 2012 (6 months); T4: Last stage of the telehealthcare program, from September 2012 to February 2013 (6 months).

## Discussion

### Principal Findings

The aims of this study were to determine how diabetic patients use online applications, determine the impact of the telehealthcare program on the patients’ 7 self-care behaviors, and examine HbA_1c_ level changes during the course of participation in such a program. Through the use of the online diabetes self-management system and the teleconsultant service, the diabetic patients managed complex information about their diseases and obtained support from their care providers. Patients were reminded to perform SMBG, and their self-care behaviors were reinforced. In all, 90% of the patients used the online diabetes self-management system, and 98% performed SMBG. On average, patients logged in every week and performed the SMBG daily. Patients who maintain insulin records are also likely to maintain dietary records, which are related to the effects of insulin injections on food intake. In this study, the use of an image uploading application did not cause more patients to maintain dietary records. One explanation for this finding is that there were more elderly patients in this program and these patients do not often use smartphones or carry a camera with them.

The case managers observed that technological difficulties were the main reason for a decline in the use of the online diabetes self-management system by elderly patients. Some of the patients claimed that they were too busy to use the online diabetes self-management system. Previous research pointed out that elderly people had poor technical skills [41]. In our study, the average age of the enrolled patients was older than 50 years; blood glucose, blood pressure, and heart rate were the 3 items most often recorded. This indicates that a proportion of the patients only used the 3G glucometer to upload data, but seldom logged into the online diabetes self-management system. Despite the technological difficulties, patients unfamiliar with information technologies were not troubled with the computer applications used in this program, and they used the 3G glucometer to participate and obtain support from their case managers in a seamless manner. Easier ways to upload data may increase the participation of those unfamiliar with technology, enhance the completeness of the dataset, and allow them to obtain support from a distance.

According to the online diabetes self-management system logged records, more patients used the phone call service than the messaging service to contact the case managers. However, the case managers found that the younger patients used text messages more often than the elderly patients did. They used various applications, such as email, smartphone applications (eg, Whatsapp, LINE), and SMS text messages, to communicate with patients in order to accommodate the patients’ busy lifestyles, which made the records difficult to trace and unavailable for presentation here. Some of the patients expressed a reluctance to let their colleagues or the people around them know about their disease, and they considered the asynchronous messages useful in allowing them to communicate with caregivers without the risk of being overheard by others. Most of the elderly patients were not familiar with keyboard typing, and they relied on phone calls to communicate. It is worth noting that providing free test strips or services created an incentive for patient participation. In fact, most of the patients were reluctant to continue their participation when told the test strips or the service may no longer be free, indicating the financial burden borne by persons with diabetes.

In the behavioral analysis, the number of SMBG assessments increased significantly in the last stage, and the patients that participated in the telehealthcare service had significantly more SMBG assessments than did those without the telehealthcare service in the 6-12 month stage. Because the telehealthcare service used was based on glucose measurements, the case managers provided support and suggestions after they observed the uploaded data. SMBG was the primary self-care ability enhanced through participation in the telehealthcare program. By performing SMBG more regularly, patients encountered problems and discussed these problems more often with the CDEs.

When the patients who participated in the telehealthcare program for 18 months were compared to those who did not, 5 of the 7 behaviors showed significant differences. Although the CDEs enhance patient education based on different considerations, patients in the telehealthcare program required less support in being active, healthy eating, and problem solving, and required more support in taking medicine and healthy coping. One explanation is that the skill of insulin injection and the overcoming of psychological obstacles still requires face-to-face interaction. Each visit was done with 1 or 2 behavior assessments, and did not cover all 7 behaviors. Some of the behaviors had not been assessed yet, and were unlikely to show differences during the 6-month period. The overall time frame concluded the 7 behaviors and also represented more time to see the changes after the education. During the last period of patient participation, the assessments of healthy coping and reducing risks were higher for those who participated in the telehealthcare program compared to those who did not. Healthy coping and reducing risks are the skills that the CDEs enhanced when the patients demonstrated that they were capable of coping with their other problems. The observation of an increase in these 2 behaviors implies that the patients were more skillful.

The mean value of HbA_1c_ of participants at 6 to 12 months improved significantly compared to baseline, and slightly declined in the last period. This result indicates that the patients experienced a “worn out” period. When the patients first entered the program, they were more conscious of their self-management behaviors because they knew that someone was watching them; as a result, they improved significantly. However, after the patients became more familiar with the service and were less anxious about the program, a slight decline was observed. The HbA_1c_ variability of each 6-month interval was significantly lower than the overall HbA_1c_ variability. The decrease of the mean HbA_1c_ value implies that patients who participated in the program improved substantially across the 18-month period, and have potentially reduced the risks of complication development with less HbA_1c_ variability [49,50]. The HbA_1c_ mean demonstrates the overall glycemic control in a 3-month period, and is limited to show the variation of glycemic control during each time interval. The behaviors affected the performance of HbA_1c_, and the change of behaviors requires time to show its effect. Therefore, there may not be differences during each 6-month range, but significant differences in the long term.

### Limitations

In this small-scale pilot study, we provided a telehealthcare program that consisted of 3G glucometers, free test strips, an online diabetes self-management system, and easy access to professional support. All the program components contributed to patient improvements, although we did not measure the individual contribution of each of the components. Unfortunately, the contribution of each of the components remains unclear; this is a limitation of this study. However, those with T1DM received free test strips from the NHI. The program did produce a small benefit of HbA_1c_ control for the T1DM patients (HbA_1c_ decreased from 7.83 to 7.74), implying that the free glucometer strips were not solely responsible for the outcomes of this program.

The mean HbA_1c_ value for the telehealthcare group during the last period dropped to 7.68 and was still considered not well controlled. This may be because of the enrollment of patients with very poor glycemic control. In addition, the therapeutic responses may require more time since HbA_1c_ is a measure of average blood glucose over the course of 3 months. The patient education in this study was based on the observations of different CDEs (3 nurses and 2 dietitians), and the evaluation result may differ from CDE to CDE. Another limitation was that before the development of the DMIS, the documentation of patient education was paper-based rather than structured in the AADE7 form. The DMIS went online in July 2011 and stabilized in August 2011; the telehealthcare program was initiated in September 2011. Hence, we were unable to obtain patients’ documentation before their entry into the telehealthcare program.

While connecting the SMBG assessment and the performance of HbA_1c_ level, it could be observed that the number of SMBG assessments increased significantly and the mean HbA_1c_ level slightly increased in the last stage of patient participation. The number of SMBG assessments may refer to patients performing more SMBG and may also refer to CDEs trying to identify patient problems through encouraging them to perform more SMBG. This study has not further explored the reason of the increasing of SMBG assessment; hence, it could not explain the reason for the increasing of SMBG assessments. Further research is needed to measure the contribution of each component of the telehealthcare program and determine how to improve patient performance when they are worn out. More work is needed to demonstrate the effect of telehealthcare on specific behaviors.

### Conclusions

This study showed that using a sophisticated technological design supported the patients with diabetes in self-management. It appears that telehealthcare is effective in enhancing blood glucose monitoring, and the patients in the program showed improvements in glycemic control. The self-care behaviors affected patient outcomes and the changes in behavior required time to show effects. Telehealthcare has a positive effect on patients with diabetes, and it may encourage more technological interventions for diabetes care.

## Multimedia Appendix 1

The detailed contents of the American Association of Diabetes Educators 7 Self-Care Behaviors (AADE7) education and the telehealthcare service.

## Multimedia Appendix 2

Patient demographics, years of participation in the shared care network.

## Multimedia Appendix 3

Patient demographics, duration of diabetes.

## Multimedia Appendix 4

Patient demographics, insulin injection frequency.

## Abbreviations

3G: third-generation mobile telecommunication
AADE7: American Association of Diabetes Educators 7 Self-Care Behaviors
AES: Advanced Encryption Standard
Ajax: Asynchronous JavaScript XML
API: application programming interface
BMI: body mass index
CCD: Continuity of Care Document
CDE: certified diabetes educator
DMIS: disease management information system
DSME: diabetes self-management education
HbA1c: glycosylated hemoglobin
HIS: hospital information system
HL7: Health Level 7
HTTPS: Hypertext Transfer Protocol Secure
IRB: institutional review board
NHI: National Health Insurance
NTUH: National Taiwan University Hospital
PHR: personal health records
SMBG: self-monitoring of blood glucose
SMS: short message service
SOA: service-oriented architecture
T1: entire duration of the telehealthcare program, from September 2011 to February 2013 (18 months)
T1DM: Type 1 diabetes mellitus
T2: initial stage of the telehealthcare program, from September 2011 to February 2012 (6 months)
T2DM: Type 2 diabetes mellitus
T3: middle stage of the telehealthcare program, from March 2012 to August 2012 (6 months)
T4: last stage of the telehealthcare program, from September 2012 to February 2013 (6 months)
XML: Extensible Markup Language
