# Supplementary material for: Evaluating Self-Management Behaviors of Diabetic Patients in a Telehealthcare Program: Longitudinal Study Over 18 Months
Source: J Med Internet Res. 2013 Dec 9;15(12):e266. doi: 10.2196/jmir.2699 (PMC3869106; doi:10.2196/jmir.2699)
Supplement: Supplementary file 1 [file jmir_v15i12e266_app1.pdf]

Appendix 1. The detailed contents of the American Association of Diabetes Educators 7 Self-Care Behaviors (AADE7) education and the telehealthcare service.

AADE7 Educations	Content of Educations	Telehealthcare Service
Being Active	Habit of exercising<br>Type of exercise<br>Frequency of exercising<br>Reason of not exercising<br>Evaluations of CDEs[a]	Monitor daily glucose and daily excise records<br>Encourage to do sufficient exercise
Healthy Eating	Intake of fiber<br>Intake of fat and cholesterol<br>Intake of high oil<br>Intake of sodium and dessert<br>Evaluations of CDEs[a]	Monitor daily glucose and daily food intake records<br>Provide adequate dietary knowledge and adjustment support
Taking Medication	Cooperation and obedience of medication order<br>Knowledge of insulin injection<br>Evaluations of CDEs[a]	Monitor daily glucose and daily insulin injection records<br>Enhance the skill of insulin injection and medication obedience
Healthy Coping	Reasons of pressure<br>Ways to relieve pressure<br>Evaluations of CDEs[a]	Provide humanity support
Problem Solving	Frequency of Hypoglycemia<br>**Ways to treat Hypoglycemia**<br>Frequency of Hyperglycemia<br>Ways to treat Hyperglycemia<br>Evaluations of CDEs[a]	Support in coping with Hypoglycemia and Hyperglycemia
Reducing Risks	Habits of Smoking<br>Foot care<br>Complication evaluation<br>Evaluations of CDEs[a]	Daily status monitoring<br>Support in foot care<br>Suggestion to quit smoking
Monitoring	Knowledge of SMBG[b]<br>Method of performing SMBG[b]<br>Frequency of SMBG[b]<br>Recording of SMBG[b]<br>Reason of not performing SMBG[b]<br>Evaluations of CDEs[a]	Enhance the skill of SMBG[b]<br>Remind of performing SMBG[b]<br>Monitor daily glucose

[a]CDE: Certified diabetes educator
[b]SMBG: Self monitoring of blood glucose
