# Supplementary material for: Evaluating Self-Management Behaviors of Diabetic Patients in a Telehealthcare Program: Longitudinal Study Over 18 Months
Source: J Med Internet Res. 2013 Dec 9;15(12):e266. doi: 10.2196/jmir.2699 (PMC3869106; doi:10.2196/jmir.2699)
Supplement: Supplementary file 2 [file jmir_v15i12e266_app2.pdf]

Appendix 2. Patient demographics, years of participation in the shared care network.

Years Entered Shared care	Patient Numbers	
	Telehealthcare (%)	Control (%)
Less than 5 years	8 (44.4)	9 (28.1)
5 years or more	10 (55.6)	23 (71.8)
T1DM[a], mean (SD)	5.17 (2.31)	6.03 (2.65)
Less than 5 years	17 (41.5)	26 (36.6)
5 years or more	24 (58.5)	45 (63.4)
T2DM[b], mean (SD)	5.46 (2.88)	5.66 (3.06)
Total mean (SD)	5.37 (2.70)	5.78 (2.93)

[a] T1DM: Type 1 diabetes mellitus
[b] T2DM: Type 2 diabetes mellitus
