# Supplementary material for: Evaluating Self-Management Behaviors of Diabetic Patients in a Telehealthcare Program: Longitudinal Study Over 18 Months
Source: J Med Internet Res. 2013 Dec 9;15(12):e266. doi: 10.2196/jmir.2699 (PMC3869106; doi:10.2196/jmir.2699)
Supplement: Supplementary file 3 [file jmir_v15i12e266_app3.pdf]

**Appendix 3**. Demographic information of patients, duration of diabetes

| Duration | | Patient Number Telehealthcare (%) | Patient Number Control (%) |
|---|---|---|---|
| T1DM[a] | < 10 | 7 (38.9) | 11 (34.4) |
| | 10 - 15 | 3 (16.7) | 8 (25.0) |
| | 15 - 20 | 5 (27.8) | 6 (18.8) |
| | > 20 | 3 (16.7) | 7 (21.9) |
| | Mean (SD) | 13.94 (8.65) | 15.16 (9.47) |
| T2DM[b] | < 5 | 3 (7.3) | 2 (2.8) |
| | 5 - 10 | 7 (17.1) | 13 (18.3) |
| | 10 - 15 | 13 (31.7) | 24 (33.8) |
| | > 15 | 18 (43.9) | 32 (45.1) |
| | Mean (SD) | 14.34 (7.25) | 15.18 (7.75) |
| Total | | 14.22 (7.63) | 15.17 (8.28) |

[a] T1DM: Type 1 diabetes mellitus
[b] T2DM: Type 2 diabetes mellitus
