# Supplementary material for: Evaluating Self-Management Behaviors of Diabetic Patients in a Telehealthcare Program: Longitudinal Study Over 18 Months
Source: J Med Internet Res. 2013 Dec 9;15(12):e266. doi: 10.2196/jmir.2699 (PMC3869106; doi:10.2196/jmir.2699)
Supplement: Supplementary file 4 [file jmir_v15i12e266_app4.pdf]

Appendix 4. Patient demographics, insulin injection frequency.

Insulin Treatment		Patient Number TELE[c] (%)	Patient Number CON[d] (%)
T1DM[a]	No[c]	0 (0.0)	1 (3.1)
	Once[d]	0 (0.0)	0 (0.0)
	Twice or more[e]	18 (100.0)	31 (96.9)
T2DM[b]	No[c]	16 (39.0)	32 (45.1)
	Once[d]	3 (7.3)	4 (5.6)
	Twice or more[e]	22 (53.7)	35 (49.3)

[a] T1DM: Type 1 diabetes mellitus
[b] T2DM: Type 2 diabetes mellitus
[c] No: No insulin injection
[d] Once: Insulin injection once per day
[e] Twice or more: Insulin injection twice or more per day
